# Amitraz Poisoning - Tale of an Unusual Pesticide Poisoning: A Case Report

**DOI:** 10.31729/jnma.4919

**Published:** 2020-05

**Authors:** Olita Shilpakar, Bipin Karki, Bibek Rajbhandari

**Affiliations:** 1 Department of General Practice and Emergency Medicine, National Academy of Medical Sciences, Bir Hospital, Kathmandu, Nepal; 2 Department of Critical Care Medicine, Om Hospital and Research Centre, Kathmandu, Nepal; 3 Department of General Practice and Emergency Medicine, Nepal Police Hospital, Kathmandu, Nepal

**Keywords:** *bradycardia*, *hypotension*, *poisoning*, *vomiting*

## Abstract

Amitraz is a formamidine group of compounds used in many parts of the world as an agricultural pesticide and an ectoparasiticide. Amitraz intoxication secondary to the oral, dermal or inhalational routes, both accidental and suicidal, has been encountered in humans leading to severe life-threatening side effects. Only very few cases of amitraz toxicity have been reported to date. This emphasizes the importance to ascertain amitraz intoxication from more commonly encountered pesticides like organophosphorus poisoning to avoid erroneous management of the patient. We report a case of a twenty-seven-year-old man who presented to the emergency room following suicidal ingestion of amitraz with the clinical manifestations of vomiting, altered sensorium, bradycardia and hypotension and complete recovery following supportive management.

## INTRODUCTION

Amitraz is a pharmacologically active compound with insecticide and acaricide properties used worldwide both on crops and animals to control pests.^1,2^ It is an alpha-2 adrenergic agonist that acts by stimulating alpha-2 adrenergic receptor sites in the central nervous system (CNS) and alpha-1 adrenergic and alpha-2 adrenergic receptor sites in the periphery. It also inhibits the activity of monoamine oxidase (MAO) and prostaglandin E2 synthesis.^3^ The patient may present with clinical features varying from nausea, vomiting, CNS manifestations like drowsiness, convulsions or coma, miosis or rarely mydriasis, hypotension, bradycardia, respiratory depression, hyperglycemia, glycosuria, hypothermia and decreased intestinal motility.^3,4^

## CASE REPORT

A twenty-seven-year-old male presented to the Emergency Room (ER) with the alleged history of ingestion of an unknown poison, amount unknown, ten hours back following a family dispute. The patient immediately had two episodes of vomiting and then gradually developed altered sensorium in the form of drowsiness within two hours of ingestion of the poison. On examination in the ER, the patient had a Glasgow coma scale score of 9/15 (E2V2M5). Vital signs revealed a radial pulse of 50 beats per minute, blood pressure 90/50mm Hg, respiratory rate 16 breaths/minute, the temperature of 97.2 degrees F and oxygen saturation of 86% in room air. His pupils were constricted bilaterally and sluggishly reacting to light. His chest auscultation revealed bilateral vesicular breath sounds, the abdomen was soft with normal bowel sounds on auscultation. Deep tendon reflexes were present with down going plantars.

The patient was provided oxygen at two litres per minute via nasal cannula, his oxygen saturation improved to 96%. He was managed by administering immediately a bolus dose of 500ml of 0.9% of normal saline and an intravenous dose of atropine 0.6mg following which his heart rate increased to 96 beats per minute showing a normal sinus rhythm in the 12 lead electrocardiogram and was normotensive. His investigations comprising a full blood count, renal function tests, a random blood sugar level, liver function tests, and a coagulation profile were all within normal limits. Arterial blood gas (ABG) analysis did not reveal any abnormalities and a serum cholinesterase level was within normal limit.

Six hours of close monitoring with continuous supportive management resulted in the patient opening his eyes spontaneously with a purposeful response to verbal stimuli. His Glasgow coma score improved to 15/15 and his symptoms resolved over the next 12 hours. During his stay, he did not develop any convulsions, respiratory depression or hypothermia. His bowels moved on the second day of admission and his urine output was normal throughout his stay. His blood glucose, measured every six hours for 24 hours and then every 12 hours, did not reveal hyperglycemia. An empty bottle of the pesticide amitraz 12.5% recovered from the patient’s room was brought along by the patient’s relative to which the patient agreed having ingested two teaspoonful of it. He was kept under observation with strict hemodynamic monitoring for the next three days which was uneventful. He was finally discharged from the hospital on the fourth day in good health.

## DISCUSSION

Amitraz is a veterinary ectoparasiticide and an agricultural product having a wide spectrum of action varying from the treatment of ticks and mites in cattle, sheep, and demodicosis in canines, keds or lice in livestock to red spider mites and psylla in fruit crops.^1,2^ Formulations of amitraz generally contain 12.5-20% of the drug in organic solvents like xylene.^3^ Toxicity usually occurs via ingestion, inhalation or even following skin exposure to the compound. Amitraz ingestion is commonly suicidal in adults, however, accidental ingestion by children has also been reported.^2,3^

Amitraz manifests its toxicity mostly with clinical features of CNS depression, respiratory depression and cardiovascular effects like hypotension and bradycardia, vomiting, miosis or mydriasis, and hyperglycemia.^3^ The toxic effects of amitraz mimic clonidine like syndrome and are the most frequently reported symptoms in this poisoning.^3,4^ Amitraz also presents with hypothermia since it has antipyretic and anti-inflammatory activities which are mostly due to the inhibition of prostaglandin E2 synthesis in vivo.^3^,^4^Organophosphorus or carbamate toxicity display several similar clinical features which could lead to a misdiagnosis.^5^

The onset of action in most case reports ranged between 30-180 minutes following ingestion. The predominant manifestation was drowsiness and CNS depression was observed within 30-90 minutes of ingestion with GCS of 9 which resolved within 8 to 14 hours in a case series by Yilmaz et al.^1^ Aydin K. et al. had described CNS depression in eight children occurring within 30-120 minutes and resolving after 8-18 hours.^2^,^6^ In our case, our patient had developed altered sensorium 90 minutes after ingestion presenting with a GCS of 9 to the emergency room and regained consciousness within 16 hours. Stupor followed by coma were attributable to the solvents like xylene and propylene oxide present in amitraz.

Miosis has been described commonly, at low doses, alpha-2 adrenergic agonists induce miosis by its action on presynaptic receptors, as encountered in our patient with bilateral pupils one millimetre in size and sluggishly reacting to light.^1^,^8^,^9^

Cardiovascular manifestations of amitraz like bradycardia and hypotension and nonspecific ECG changes have been reported in many case reports, the major cause being the action of alpha-1 and alpha-2 agonists.^6^,^7^ A near-fatal case of amitraz and xylene poisoning has been reported by Vucinic et al. showing atrial fibrillation in the ECG.^7^ A 12 lead ECG in our case showed sinus bradycardia only. A few cases of severe respiratory depression requiring elective intubation and mechanical ventilation have also been reported.^1^,^7^,^9^

The haematological parameters, renal functions, and liver function tests do not alter in most of the cases like in our case. Glycosuria due to the action of amitraz on alpha-2 adrenergic receptors in pancreatic islets inhibiting insulin thus stimulating glucagon secretion has been reported in rats. Likewise, hyperglycemia has also been reported in humans, however, our patient maintained a normoglycemic level throughout his stay at the hospital.^7^,^8^ An arterial blood gas analysis showed no abnormality in our patient, however, metabolic acidosis in five cases and respiratory alkalosis in two cases have been reported by Kalyoncu and colleagues in a case series.^8^

No antidote is available for amitraz poisoning to date, so the mainstay of treatment is essentially symptomatic. It includes hemodynamic stabilization with intravenous fluid resuscitation, airway management, oxygen administration, reduction of absorption and elimination of the poison from the body. There is no data regarding the role of activated charcoal or gastric lavage concerning amitraz poisoning to date. Our case did not require inotropic support at any point in management for hypotension, indicating that these effects may be dose-dependent and intravenous fluid resuscitation was the mainstay of treatment for hypotension in our case. In a case report from Turkey, the patients responded to intravenous fluids and atropine sulphate administration alleviated bradycardia which was similar to our case report.^2^ However, several cases reports have mentioned IV fluids as well as inotropic support to counteract hypotension.^9,10^ The toxic effects usually last for 24 hours with the patients recovering well after symptomatic management and is rarely fatal in humans.

Despite life-threatening symptomatology, amitraz poisoning may recover well with initial stabilization, close monitoring, and supportive treatment. Our patient had a relatively quick and satisfactory recovery, his condition improving after 12 hours of amitraz ingestion. Yohimbine, an alpha-2 adrenergic antagonist has been tried on animals to reverse the effects of amitraz.^9,10^ However, further studies focusing on the evaluation of the efficacy and safety of these agents in antagonizing amitraz toxicity is recommended. We would also like to emphasize the importance of public education on primary prevention of poisoning, awareness among the physicians regarding mimickers like organophosphates and precautionary measures like redesigning of containers with warning labels.
